# The Inhibition Effect of Cell DNA Oxidative Damage and LDL Oxidation by Bovine Colostrums

**DOI:** 10.3390/molecules21101378

**Published:** 2016-10-21

**Authors:** Chih-Wei Chen, Chi-Yue Chang, Shu-Hua Chiang

**Affiliations:** 1 Department of Health Food, Chung Chou University of Science and Technology, Changhua 51591, Taiwan; ccwlly@gmail.com (C.-W.C.); charles0201@dragon.ccut.edu.tw (C.-Y.C.); 2 Department of Health and Creative Vegetarian Science, Fo Guang University, No. 160, Linwei Rd., Jiaosi, Yilan County 26247, Taiwan

**Keywords:** bovine colostrum, DNA oxidative damage, low density lipoprotein (LDL), LDL oxidation

## Abstract

In the present study, we investigated the effect of bovine colostrums on inhibition of DNA oxidative damage and low density lipoprotein (LDL) oxidation in vitro. Results showed that whey and skimmed milk exhibited not only higher inhibitory activities of oxidative damage of deoxyribose but also an inhibitory effect on the breakdown of supercoiled DNA into open circular DNA and linear DNA. The quantities of 8-OH-2′-dG formed under whey, caseins and skimmed milk treatment were 0.24, 0.24 and 1.24 μg/mL, respectively. The quantity of malondialdehyde formed through LDL oxidation induced by copprous ion was significantly decreased as colostrums protein solutions were added, in which whey and caseins led to a more significant decrease than skimmed milk. The formation of conjugated dienes could be inhibited by treatment with colostrums protein solutions. Whey exhibited the longest lag time of conjugated dienes formation among the colostrums proteins. The lag time of the whey was 2.33 times that of the control. From the results of foregoing, the bovine colostrums protein has potential value in the inhibition of DNA oxidation damage and LDL oxidation.

## 1. Introduction

DNA, as the repository of genetic information in living cells, is remarkably susceptible to damage induced by exogenous or/endogenous factors [1]. In recent years, the damage degree of DNA induced by reactive oxygen species (ROS), such as hydroxyl radicals (•OH) hydrogen peroxide (H_2_O_2_) and superoxide (O_2_^−^) or the capacity of antioxidant for scavenging ROS has been widely investigated in the food technology and human health fields [1,2]. Several research papers indicate that oxidized low-density lipoprotein (LDL) within the arterial wall promotes the development of atherosclerosis [3]. Protection against LDL oxidation is an effective strategy to prevent atherosclerosis [3,4], and growing evidence from epidemiologic studies has shown that dietary antioxidants contribute to the prevention of coronary heart disease [3,5]. Many antioxidants have an effect in the inhibition of lipid oxidation. However, they fail to protect these matrices, such as DNAs, carbohydrates and proteins from oxidative damage. For example, while butylatedhydroxyanisole can effectively retard peroxidation of lipid, it is expected to result in the affection of pre-carcinoma of stomach in rats by an excessive intake, which is attributed to oxidative damage of DNA [6]. Therefore, efforts in searching for a bioavailable antioxidant to terminate chain reactions of radical and to inhibit the oxidation of DNA in the human body has become critical.

Bovine colostrums is the first milk produced postpartum and is typically defined as the first six postpartum milking collected during the period of transition from colostrums to milk [7,8]. Several researchers have compared the composition of colostrums with those of mature milk and concluded that colostrums have higher levels of protein, lower levels of fat, a lactose solution rich in immunoglobulins, and other important immune elements and mediators [9,10,11]. Bovine colostrums are not only rich in cytokines, including interferon-γ (IFN-γ), interleukin (IL) and tumor necrosis factor-α (TNF-α) but also contain essential nutrients such as proteins, carbohydrates, lipids and vitamin, which are produced in the mammary glands and secreted after parturition [12]. Colostrums play a key role in the transmission of necessary nutrients, growth factors and immunological components from mother to neonate.

In this study, bovine colostrums were collected on the second postpartum day and used the preparation of skimmed milk, casein, and whey proteins to investigations the antioxidation of bio-molecules, defending DNA oxidative damage induced by hydrogen peroxide, and protecting oxidative modification of LDL. We focus on the compositions of colostrums, their defending effect regarding DNA oxidative damage and their concentration partition regarding the protective effect of LDL oxidative modifications.

## 2. Results

### 2.1. Effect of Bovine Colostrums Protein on the Fenton Reaction-Induced Oxidative Damage of Deoxyribose

**Figure 1:**
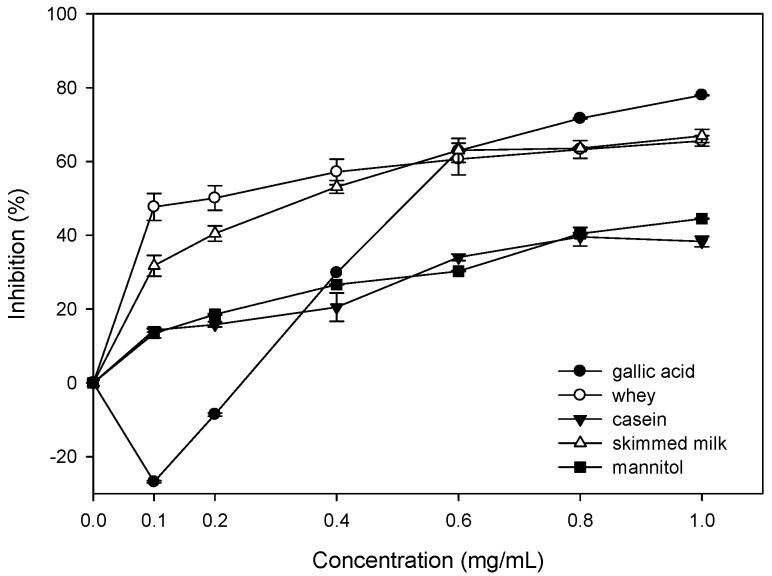
Effect of whey, casein and skimmed milk on the Fe^2+^-ethylenediaminetetraacetic acid (EDTA)/H_2_O_2_/Asc induced oxidative damage of deoxyribose.

The result indicates that the bovine colostrums protein has a significant inhibition against the oxidation damage on deoxyribose (Figure 1). Meanwhile, the inhibition rate goes up with the increasing sample concentration. When the concentration reaches 0.6 mg/mL, the inhibition rate of three proteins become stable. The whey protein solution has an inhibition rate of 47.6% at 0.1 mg/mL. In contrast, the gallic acid shows oxidation promotion when the concentration is below 0.2 mg/mL. It is reported that the gallic acid can clean the O_2_^−^, HOCl, and inhibits the peroxidation of liposome [13]. However, it promotes oxidation in the oxidation damage system in deoxyribose induced by Fenton reaction. The concentration accords with the low level adopted in this experiment. The oxidative stability improves with increasing concentration. When the concentration is 1.0 mg/mL, the inhibition rate has reached 77.9%. That accords with Hsieh and Yen (2000) [14], which is 79% at 1.14 mg/mL. When the concentration is 1.0 mg/mL, the inhibition rates of whey, casein, and skimmed milk are 65.6%, 38.3%, and 66.9%, respectively. The result indicates that the bovine colostrum protein does not promote any oxidation. In contrast, it is a good •OH cleaner. Whey and skimmed milk demonstrate very good inhibition effects.

### 2.2. Effect of Bovine Colostrums Protein on DNA Single-Strand Cleavage Induced by Fenton Reaction

**Figure 2:**
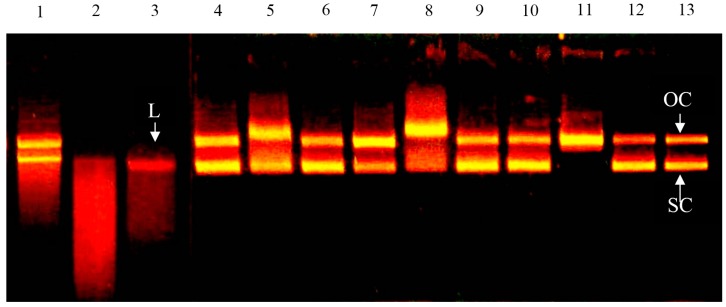
Effect of bovine colostrums protein on DNA single-strand cleavage induced by Fe^2+^ and Fenton reaction. Phage ψX174 DNA supercoiled DNA (0.3 μg) was incubated with Fe^3+^ (lane 13), lanes 1–3: 0.1, 1, 10 mg/mL ascorbic acid, lanes 4–6: 0.1, 1, 10 mg/mL whey, lanes 7–9: 0.1, 1, 10 mg/mL casein, lanes 10–12: 0.1, 1, 10 mg/mL skimmed milk.

Figure 2 shows that in the control group (lane 13) with only ferrous ions added, both supercoiled and open circular DNA can be found. There are free radicals generated in the Fenton reaction from supercoiled DNA. Some of the supercoiled DNA is cut into open circular DNA. This is similar to the results of Kobayashi et al. (1990) [15]. The concentration is 0.1 mg/mL (lanes 4, 7, 10), the bovine colostrum protein solutions have no significant impact on the damage by •OH. When the concentration is 1 mg/mL (lanes 5, 8, 11) the oxidation promotion can be observed in all solutions, and when the concentration is 10 mg/mL (lanes 6, 9, 12) the oxidation promotion disappears. The results showed that under a low concentration, the bovine colostrums protein solution has a limited ferrous ion chelating ability and cannot finish the ferrous ion chelating effectively. The reduction of oxidation damage is because of the chelating capacity, which increases when the concentration increases. For the ascorbic acid, (lanes 1–3), oxidation promotion can be observed in all concentrations, especially when the concentration is 1 mg/mL (lane 2). Worse DNA oxidation damage can be observed. As a result, no linear DNA can be seen and there are some DNA segments left.

### 2.3. Effect of Bovine Colostruns Protein on the Oxidation of 2′-dG to 8-OH-2′-dG Induced by Fenton Reaction

In Table 1, it is shown that no significant difference can be found there under different concentrations by whey, casein, and skimmed milk. Compared to ascorbic acid, these solutions do not promote the generation of 8-OH-2′-dG under any circumstances. The output of skimmed milk is a bit higher than the whey protein and casein. A possible reason for this is that the bovine colostrums protein does not have a strong reducing capacity. Free radicals will not oxidize the 2′-dG continuously.

### 2.4. Effect of Bovine Colostrums Protein on Bleomycin-Dependent DNA Damage

**Figure 3:**
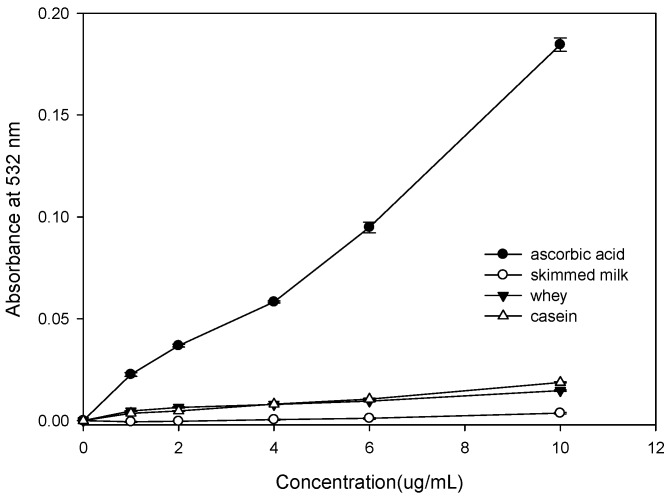
Effect of bovine colostrum on DNA damage induced by bleomycin-Fe^3+^.

Figure 3 shows that under 10 μg/mL, the ascorbic acid promotes the oxidation damage of DNA by bleomycin-Fe^3+^ significantly. A major reason for this is that the ascorbic acid reduces the Fe^3+^ into Fe^2+^. The Fe^2+^ reacts with H_2_O_2_ and generates •OH. Free radicals result in DNA damage [16]. The absorbance of whey, casein, and skimmed milk does not increase with concentration in this system. A possible reason for this is that the bovine colostrums proteins are capable of cleaning •OH and Fe^2+^ chelating. Meanwhile, none of them have the capacity to reduce the Fe^3+^ and protect the DNA from oxidation damage by free radicals.

### 2.5. Inhibition of Oxidative Damages of Biomolecules by Bovine Colostrums Protein

The inhibition rates of DNA oxidation damage that are induced by bleomycin-Fe^3+^/Asc, are 8.41%, 6.62% and 2.33%, respectively by treatment with whey, casein and skimmed milk (Table 2). The inhibition rates of 8-OH-2′-dG generation from 2′-dG induced by Fe^2+^-EDTA/H_2_O_2_/Asc, are 20.01%, 18.18%, and 11.07%, respectively (Table 2).

### 2.6. Effects of Bovine Colostrums Protein on the Formation of TBARS and Conjugated Dienes on LDL Oxidation Induced by Cu^2^^+^

**Figure 4:**
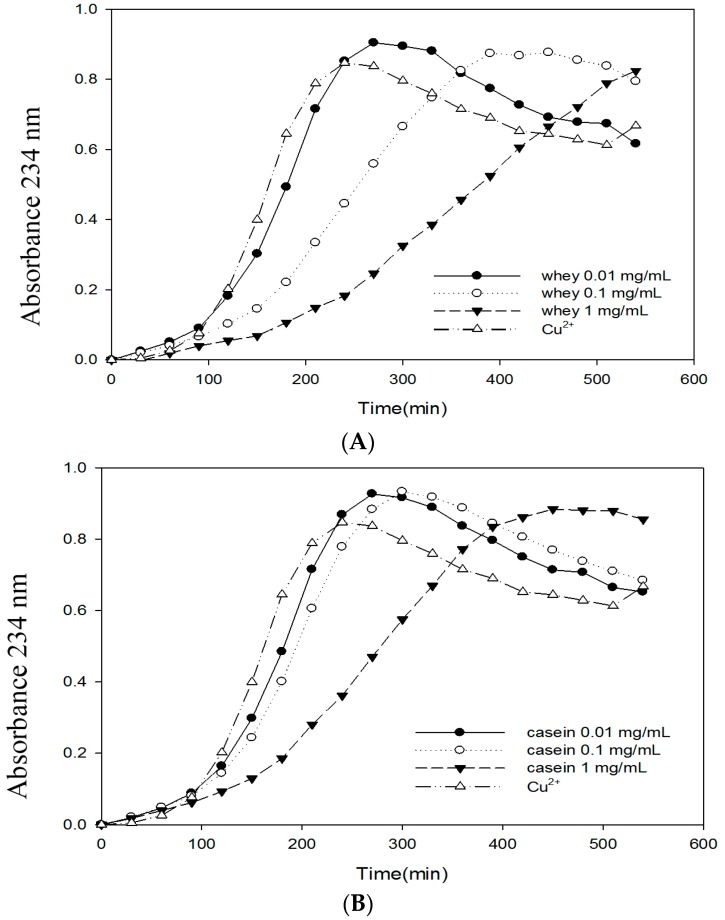

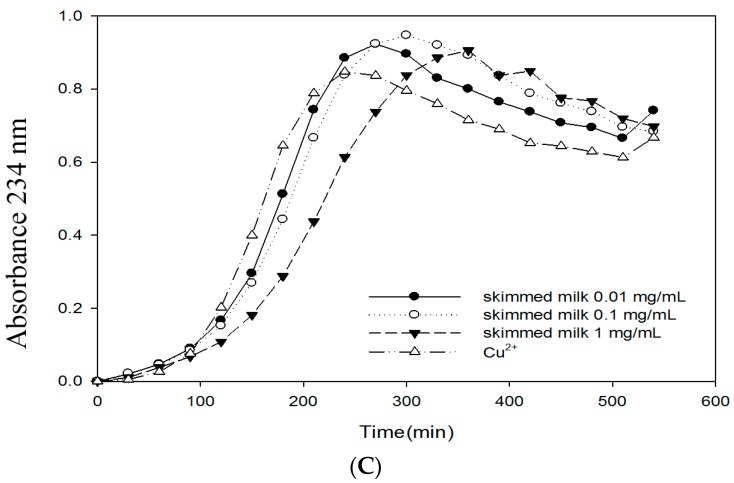
Effects of bovine colostrums protein on Cu^2+^ mediated conjugated diene formation in LDL. LDL (100 μg protein/mL) was incubated with 10 μM CuSO_4_ at 37 °C in the absence or presence of bovine colostrums protein. Conjugated diene was used to measure the absorbance at 234 nm every 30 min for 540 min and the results express relative absorbance at 234 nm. (**A**): Whey; (**B**): Cassin and (**C**): Skimmed milk.

Table 3 shows the protection of bovine colostrums protein against LDL oxidation damage. The results showed that when the concentration is 1 mg/mL, both whey and casein have good oxidative stabilities. The TBARS outputs are 4.07 and 4.18 nM/mL respectively. Both of them are significantly lower than the control group. Meanwhile, the TBARS output tends to increase with decreasing concentration. Table 3 also shows the effects of bovine colostrums protein on the formation of conjugated diene from LDL oxidation induced by Cu^2+^. Only LDL and Cu^2+^ are added into control group, whose lag time is 90 min. When the concentration is 0.01 mg/mL, the lag time for all groups is quite close to that of the control group. When the concentration is 0.1 mg/mL, the lag time for whey protein extends to 150 min (Figure 4). No significant extension is found for the rest of the groups. When the concentration is 1 mg/mL, significant inhibition can be observed. The lag times for whey, casein, and skimmed milk were 210, 150, and 120 min, respectively. They are 2.33, 1.66, and 1.33 times that of the control group.

## 3. Discussion

The first study tries to discuss whether the bovine colostrums protein can be used to limit the oxidation damage of deoxyribose induced Fe^3+^-EDTA/H_2_O_2_/Asc system. Figure 1 demonstrates the inhibition against the Fenton reaction induced oxidation damage of deoxyribose by whey, casein, and skimmed milk. Since the deoxyribose will break into malondialdehyde by the attack of hydroxyl free radicals, the formation of one of the products, malondialdehyde (MDA), forms the basis of the deoxyribose assay. Damage to the sugar parts of the DNA structure can lead to breaks in the strands of DNA, because these constitute the phosphate–deoxyribose backbone [17]. When the concentration is 1.0 mg/mL, the inhibition rates of whey, casein, and skimmed milk are 65.6%, 38.3%, and 66.9%, respectively. The result indicates that the bovine colostrum protein does not promote any oxidation. On the contrary, it is a good •OH cleaner. Whey and skimmed milk demonstrate very good inhibition effects. This also proves that none of them has obvious reducing power [18]. In a study on the extractions of eucommia bark, eucommia bark leaf, fried and fresh eucommia barks have inhibition effects of 85%, 68%, and 49%, respectively. That accords with our result. Furthermore, the inhibition rate tends to increase with the concentration increase [14]. Although the reducing power plays an important role in oxidative stability, it is even more important in oxidation promotion, especially when there is a transition metal. The reducibility may promote the oxidation damage of the biological molecules [19]. Strong reducing power can reduce the Fe^3+^ into Fe^2+^ and increase the formation of •OH. That is why the gallic acid fails to keep the Fe^2+^ away from the biological molecules, together with its bad Fe^2+^ chelating. As a result, the Fe^2+^ cannot be controlled and there are site-specific oxidation damages on biological molecules [14].

ψX174 RF I is a supercoiled double strand DNA. If a single strand of ψX174 RF I DNA is broken, it switches from type RF I into type RF II, which is not supercoiled. The supercoiled architecture is then turned into open circular. The supercoiled DNA has a good electrophoretic fluidity in colloid. The double strand structure may break into open circular DNA, which will lower the electrophoretic fluidity. Linear DNA has an electrophoretic fluidity between supercoiled and open circular DNA [20]. Figure 2 shows the ascorbic acid, (lanes 1–3), oxidation promotion can be observed in all concentrations, especially when the concentration is 1 mg/mL (lane 2). Worse DNA oxidation damage can be observed. As a result, no linear DNA can be seen and there are some DNA segments left. The study pointed out that with 5 mM (0.88 mg/mL) ascorbic acid, the DNA can be cut into drags. That accords with our results [21]. Although the ascorbic acid and polyphenol compounds can be used to control the overoxidation of the lipids, it is found that these substances are oxidation-promotional. For example, the ascorbic acid can prevent the cells from oxidation damages, but if Fe^3+^ or Cu^2+^ are present, generation of reactive oxygen species (ROS) will be promoted [22]. They also promote the generation of 8-OH-2′-dG in mouse′s cystoblast [23].

8-OH-2′-dG turns out to be the most significant biological index of DNA oxidation damage in both internal and external experiments [24,25]. At the same time, it has the potential of mutagenicity [26]. Therefore, measuring the content of 8-OH-2′-dG can be used to evaluate the oxidation pressure in the body [27]. According to the results (Table 1), massive generation of 8-OH-2′-dG is impossible. Yen et al. (1997) [28] pointed out that adding ascorbic acid into the system with similar concentration can result in the transformation from 2′-dG into 8-OH-2′-dG. That accords with the results of this experiment. In a study on eucommia bark extraction, its 8-OH-2′-dG output was higher than that of bovine colostrums protein [14].

Bleomycin is a member of the glycopeptides family. It is a kind of antibiotic containing several rare amino acid and carbohydrate molecules. The type differs based on the end amino acid. Bleomycin is a mixture [29] of Bleomycin adopted in a clinic and includes 55%–70% A2, 25%–32% B2, A2′ (<7%), and 1% B4. Its cell base looks radial because of toxic effects. When there is Fe^2+^ and oxygen, the DNA will be destroyed and the thymine will become dissociative, or the DNA structure will be broken [30]. Figure 3 shows that the absorbance of bovine colostrums protein does not increase with concentration. A possible reason for this is that the bovine colostrums proteins are capable of cleaning •OH and Fe^2+^ chelating.

From the results of 8-OH-2′-dG generation and DNA oxidation damage induced by bleomycin-Fe^3+^ system, the ascorbic acid may result in significant oxidation damage with the existence of Fe^3+^. In this experiment, add the ascorbic acid into the reaction system first and wait for the oxidation damage. After that, add bovine colostrums protein into it. Then, compare its inhibition to the ascorbic acid and calculate the inhibition rate. For the impacts of whey, casein, and skimmed milk on DNA oxidation damage induced by bleomycin-Fe^3+^/Asc, the inhibition rates are 8.41%, 6.62% and 2.33%, respectively (Table 2). With the exception of bovine colostrums protein, significant inhibitions were demonstrated. For the impacts of whey, casein, and skimmed milk on 8-OH-2′-dG generation from 2′-dG induced by Fe^2+^-EDTA/H_2_O_2_/Asc, the inhibition rates are 20.01%, 18.18%, and 11.07%, respectively (Table 2). Whey and casein are better than skimmed milk in 8-OH-2′-dG inhibition. Chiang and Chang (2005) [18] reported that lactoferrin have strong antioxidant activity such as reducing power, Fe^2+^ chelating and free radical cleaning ability; the contents of lactoferrin was in order to whey > casein > skimmed milk. Based on the above results, we come to the conclusion that the inhibition against oxidation damage on biological molecules is likely to be: whey > casein > skimmed milk. Low Fe^2+^ chelating and free radical cleaning are reasons for low inhibition of skimmed milk [18].

The TBARS output tends to increase with decreasing concentration (Table 3). For the skimmed milk, the TBARS output will be 4.72 nM/mL when the concentration is 1 mg/mL. That is quite similar to the control group. In the studies, the fluorescent measurement is implemented after 24 h of Cu^2+^ reaction. The skimmed milk is low in chelating [18]. Excessive metal ions lead to more TBARS generation. That is why there are more TBARS outputs.

## 4. Materials and Methods

### 4.1. Materials

The bovine colostrums used in this study were collected from a cow on its second postpartum day at the Chu-En Ranch, Hsiushui, Changhua, Taiwan.

### 4.2. Preparation of Skimmed Milk, Caseins and Whey Proteins

The colostrums were collected at approximately 8 a.m. and promptly centrifuged at 10,000× *g* for 30 min at 4 °C to remove fat. Subsequently, the colostrums were adjusted to pH 4.6 using 1.0 N HCl and kept in a water bath at 30 °C for 30 min to complete the precipitation of caseins. The supernatant thus obtained was adjusted to pH 7.0, using 1.0 N NaOH and centrifuged again at 10,000× *g* for 30 min at 4 °C. The final supernatant after the two centrifugations was treated as a whey protein. The skimmed milk, caseins and whey proteins were freeze dried and stored at −20 °C until used.

### 4.3. Effect of Bovine Colostrums Proteinon Deoxyribose Damage (Fenton Reaction)

To test the ability of the sample solutions of bovine colostrums protein to inhibit oxidative damage of deoxyribose, the Fenton reaction model system, which contained FeCl_3_-EDTA and H_2_O_2_, was used with method of Smith et al. (1992) [16]. The reaction mixture (3.5 mL), which contained the sample (0.1–1.0 mg/mL), deoxyribose (3 mM), H_2_O_2_ (1 mM), potassium phosphate buffer (20 mM, pH 7.4), FeCl_3_(50 μM), and EDTA (100 μM), were incubated at 37 °C for 1 h with the addition of ascorbic acid (100 μM). The extent of deoxyribose degradation was measured using the TBARS method (Moran et al., 1997) [31]. One milliliter of 1% TBA and 1 mL of 2.8% TCA were added to the mixture, which was then heated in a water bath at 100 °C for 20 min. The absorbance of the resulted solution was measured by spectrophotometer at 532 nm. The control sample did not have bovine colostrums protein solutions.

### 4.4. Effect of Bovine Colostrums Protein on DNA Damage (Fenton Reaction)

To study the effect of bovine colostrums protein on the Fenton-induced oxidative breakage of supercoiled DNA to open circular DNA, DNA electrophoresis was employed using the method of Kobayashi et al. (1990) [15] with modification. Bovine colostrums protein samples (0.1, 1, 10 mg/mL), 0.3 μL of DNA (1 μg/μL), 0.1 M sodium phosphate buffer (pH 7.4), FeCl_2_ (50 μM) were mixed in a plastic tube to get a final volume of 10 μL. The mixture was incubated at 37 °C for 1 h. The reaction was stopped by the addition of 5 μL of 0.1 M EDTA containing 50% (*w/v*) sucrose and 0.1% bromophenol blue, and the solution was subjected to electrophoresis in 0.7% agarose gels with 40% mMTris-HCl/5 mM sodium acetate/1 mM EDTA as the running buffer. The gel was stained with ethidium bromide (0.05 mg/L), viewed and photographed on a transilluminator. A scanner (ARTEC, Taichung, Taiwan) scanned the photographic negatives in order to quantify the relative amount of DNA in each band, using the ID Image Analysis Software (version 2.02, Kodak Digital Science, Rochester, NY, USA).

### 4.5. Effect of Bovine Colostrums Protein on Oxidation of 2′-Deoxyguanosine (Fenton Reaction)

The effects of bovine colostrums protein on the oxidation of 2′-deoxyguanosine (2′-dG) to 8-hydroxy-2′-deoxyguanosine (8-OH-2′-dG) were assayed, using the method of Yen et al. (1997) [28] with modification. The reaction mixture (1.4 mL), containing bovine colostrums protein samples (1, 2, 4, 6, 8, 10 mg/mL), 2′-dG (0.5 mM), and potassium phosphate buffer (20 mM, pH 7.4), was initiated using the Fenton reaction model system (H_2_O_2_ (50 mM), FeCl_3_ (1.3 mM), and EDTA (6.5 mM)) with the addition of ascorbic acid (15 mM). The entire mixture was incubated at 37 °C for 30 min, and incubation was terminated by placing the samples in an ice-bath, and then filtered through a 0.45 μm filter before use. The filtrate was analyzed by HPLC (Hitachi, Tokyo, Japan), using a LiChrosphere RP-18 column (150 mm × 4 mm, 5 μm) and UV detector (measured at 254 nm). The mobile phase contained 6.5% methanol in 50 mM phosphate buffer, and the flow rate was 0.5 mL/min. 2′-dG and 8-OH-2′-dG were identified through comparison of their retention times with those of known standards, and the amount of 8-OH-2′-dG were determined based on the peak areas in the chromatograms.

### 4.6. Effect of Bovine Colostrums Protein on Bleomycin-Dependent DNA Damage

The influence of bovine colostrums protein on bleomycin-dependent DNA damage was determined according to the method of Aruoma et al. (1993) [13]. A solution (3.5 mL), which contained bovine colostrums protein samples (1, 2, 4, 6, 10 mg/mL), calf thymus DNA (0.2 mg/mL), bleomycin (0.05 mg/mL), potassium phosphate buffer (20 mM, pH 7.4), FeCl_3_ (25 μM), and MgCl_2_ (5 mM), was incubated at 37 °C for 1 h with or without the addition of ascorbic acid (240 μM). A portion (0.1 mL) of EDTA (100 mM) was added to the mixture, which was then measured using the TBA method as described above for the assay of deoxyribose damage.

### 4.7. Inhibition of Oxidative Damages of Biomolecules by Bovine Colostrums Protein

To assess the effect of colostrum proteins on the inhibition of oxidative damages of biomolecules, two experiments were designed, one for the oxidation of 2′-dG induced by Fenton reaction and the other for the oxidation of DNA induced by bleomycin-Fe^3+^ (1.5 mM) and ascorbic acid (10 μg/mL) were applied in the reaction solutions specified in Section 4.5 and Section 4.6 in the beginning of reaction, respectively, as the simulator of oxidative damages. Upon half way of the reaction (10 mg/mL) and bovine colostrums protein (10 μg/mL) were added to retard the oxidation reaction, respectively. The resulted reaction solutions were then analyzed at the end of reaction using the same procedures as described in Section 4.5 and Section 4.6.

### 4.8. LDL Preparation

Fasting plasma, for LDL isolation, was collected from normal human volunteers in tubes containing ethylenediaminetetraacetic acid (EDTA; 1 mg/mL). LDL (100 μg protein/mL) was isolated by sequential ultracentrifugation using a Hitachi ultracentrifuge (Himac CS 120GX, Hitachi) as described by Yamanaka et al. (1997) [32] with a minor modification. LDL solution was flushed with N_2_ gas, stored at 4 °C, and used within 1 week after preparation. Protein was measured using a Bio-Red kit, with bovine serum albumin as a standard. For oxidation experiments, LDL was dialyzed three times against 1 L (1000-fold volume) of phosphate buffered saline (PBS, containing 0.01 M phosphate-buffer and 0.15 M NaCl, pH 7.4) in the dark at 4 °C for 24 h.

### 4.9. LDL Oxidation

Dialyzed LDL (100 μg protein/mL) was diluted in 10 mM PBS and incubated at 37 °C in the presence or absence of 10 μM CuSO_4_. Oxidation was performed with or without the sample solution of colostrum proteins. After incubation, lipid peroxidation of the LDL was measured as described below. Ascorbic acid oxidation was used for reference.

### 4.10. Thiobarbituric Acid Reactive Substances (TBARS)

TBARS were measured using the method described by Yagi (1989) [33]. LDL solution (0.10 mg protein/mL) was added with various concentrations of colostrums proteins specified as 0.001, 0.01, 0.1, and 1.0 mg/mL. After 1 h standing in room temperature, mixture containing Cu^2+^ solution (10 mM) was added and the solution stood for another 24 h in a water bath at 37 °C for completion of reaction. At the end, 0.2 mL reaction solution was taken and added with 0.2 mL TCA (20% *w/v*) and 0.2 mL TBA (0.67% *w/v* dissolved in 0.3% NaOH solution). After thorough mixing of the reaction solution, it was heated in a water bath at 90–95 °C for 45 min. A spectrofluorometer (Hitachi F-3010) was applied to determine Ex/Em: 532 nm/600 nm. Using 1,1,3,3-Tetramethoxypropane as standard for calibration curve, the content of TBARS could be calculated.

### 4.11. Conjugated Diene

Conjugated diene formation was measured by determining the absorbance increase at 232 nm of the solution of LDL (100 μg protein/mL) in PBS incubated with CuSO_4_ (10 μM) in the absence or presence of various concentrations of bovine colostrums protein (0.01, 0.1 and 1 mg/mL). The absorbance was measured every 30 min for 540 min using a Hitachi U-2000 spectrophotometer, and the results were expressed as relative absorbance at 234 nm. The duration of the lag phase was calculated by extrapolating from the propagation phase.

### 4.12. Statistics

Results in this study were analyzed by One-way ANOVA, Correlation, and Stepwise regression functions in SAS (SAS, 2001).

## 5. Conclusions

The results indicate that bovine colostrums protein is not pro-oxidants. The decreasing sequence of inhibitive ability on oxidative damage of biomolecules is whey > caseins > skimmed milk. Recent reports on the clinical inspection of carcinoma, AIDS, and pneumonia have shown more and more specific evidence of its healthy efficacy by dietary intake of whey protein [34,35,36]. Although few researches have investigated the effect of whey protein on the body composition of humans, whey proteins have been demonstrably shown to promote glutathione content in various cells [37]. The reports regarding the treatment benefits of using bovine colostrum from antioxidant activity and composition were not substantial. Therefore, at present, results are based on the lactoferrin contents in bovine colostrums as an index of antioxidant activity: Chiang and Chang (2005) [18] reported that lactoferrin have strong antioxidant activity such as reducing power, Fe^2+^ chelating and free radical cleaning ability; the contents of lactoferrin was in the order whey > casein > skimmed milk. The lactoferrin contents of whey were significantly higher than casein and skimmed milk. In the present study, results showed that bovine colostrums protein exhibited not only higher inhibitory activities of oxidative damage of deoxyribose but also inhibitory effect on the breakdown of supercoiled DNA into open circular DNA and linear DNA. The quantities of 8-OH-2′-dG formed under whey, caseins and skimmed milk treatment were 0.24, 0.24 and 1.24 μg/mL, respectively. The quantity of malondialdehyde formed through LDL oxidation induced by copprous ion was significantly decreased as bovine colostrums protein were added, in which whey and caseins had more significant decrease than skimmed milk. The formation of conjugated dienes could be inhibited by treatment with colostrums protein solutions. Whey exhibited the longest lag time of conjugated dienes formation among the colostrums proteins. The lag time of the whey was 2.33 times that of the control. From the results of foregoing, the bovine colostrums protein has the potential value at the inhibition of DNA oxidation damage and LDL oxidation. However, the mechanism remains unclear. Further investigations using clinical or animal models would be worthwhile.

## Figures and Tables

**Table 1:** Effect of bovine colostrums protein on the oxidation of 2′-dG to 8-OH-2′-dG induced by Fenton reaction.

Addition to RM *	8-OH-2′-dG (μg/mL)		
	Whey	Casein	Skimmed Milk
PBS buffer	0.36 ^e,^**	0.36 ^b,^**	0.36 ^d,^**
15 mM Ascorbic acid	10.03 ^a^	10.03 ^a^	10.03 ^a^
1 mg/mL	0.46 ^b,c^	0.33 ^b^	1.45 ^b,c^
2 mg/mL	0.33 ^d^	0.24 ^b^	1.46 ^b,c^
4 mg/mL	0.35 ^c,d^	0.32 ^b^	1.38 ^b,c^
6 mg/mL	0.24 ^d^	0.38 ^b^	1.24 ^c^
8 mg/mL	0.55 ^b^	0.38 ^b^	1.36 ^b,c^
10 mg/mL	0.33 ^d^	0.41 ^b^	1.49 ^b^

* RM (reaction mixture) containing 0.5 mM 2′-dG, 1.3 mM FeCl_2_, 50 mM H_2_O_2_, 6.5 mM EDTA, 15 mM ascorbic acid, and 0.1 M phosphate buffer (pH 7.4) was shaken at 37 °C for 30 min. ** Values with different superscripts are significantly different (*p* < 0.05).

**Table 2:** Effect of bovine colostrums protein on the DNA damage induced by bleomycin-Fe^3+^/Asc and oxidation of 2′-dG to 8-OH-2′-dGinduced by Fe^2+^-EDTA/H_2_O_2_/Asc.

Addition to RM *	Absorbance at 532 nm	Inhibition (%)	8-OH-2′-dG (μg/mL)	Inhibition (%)
Ascorbic acid	0.186 ^a,^**		10.13 ^a,^**	
Whey	0.171 ^c^	8.41	8.12 ^c^	20.01
Casein	0.174 ^b^	6.62	8.29 ^c^	18.28
Skimmed milk	0.182 ^a^	2.33	9.02 ^b^	11.07

* RM (reaction mixture) for DNA damage: containing 0.05 mg/mL bleomycin, 25 μM FeCl_3_, 5 mM MgCl_2_, 0.2 mg/mL calf thymus DNA, 30 mM phosphate buffer (pH 7.4) and 10 μg/mL ascorbic acid was shaken at 37 °C for 30 min, then reacted with 10 mg/mL whey, casein and skimmed milk for 30 min. * RM (reaction mixture) for 2′-dG to 8-OH-2′-dG: containing 0.5 mM 2′-dG, 1.3 mM FeCl_2_, 50 mM H_2_O_2_, 6.5 mM EDTA, 15 mM ascorbic acid, and 0.1 M phosphate buffer (pH 7.4) was shaken at 37 °C for 30 min. ** Values with different superscripts are significantly different (*p* < 0.05).

**Table 3:** Effects of bovine colostrums protein on the formation of Thiobarbituric Acid Reactive Substances (TBARS) and conjugated dienes on low density lipoprotein (LDL) oxidation induced by Cu^2+^.

Concentration (mg/mL)	Whey	Casein	Skimmed Milk	Whey	Casein	Skimmed Milk
	TBARS (n mol/mL)			Lag Time * (min)		
Blank	5.13 ± 0.01 ^a,^*	5.13 ± 0.01 ^a^	5.13 ± 0.01 ^a^	90–120	90–120	90–120
1 mg/mL	4.19 ± 0.01 ^b^	4.07 ± 0.06 ^b^	4.72 ± 0.05 ^a^	180–210	150–180	120–150
0.1 mg/mL	4.13 ± 0.05 ^b^	4.21 ± 0.09 ^b^	4.80 ± 0.14 ^a^	120–150	90–120	90–120
0.01 mg/mL	4.42 ± 0.06 ^b^	4.53 ± 0.01 ^b^	4.72 ± 0.05 ^a^	90–120	90–120	90–120
0.001 mg/mL	4.98 ± 0.02 ^a^	4.85 ± 0.05 ^a^	5.02 ± 0.06 ^a^	-	-	-

* Conjugated diene formation was measured by determining the absorbance at 234 nm every 30 min for 540 min. Each value is expressed as mean ± SE (*n* = 3). Means with different letters within a row are significantly different (*p* < 0.05).
